# Biliary HMGB1 levels and biochemical indices in the assessment of acute obstructive septic cholangitis combined with septic shock

**DOI:** 10.1016/j.clinsp.2025.100611

**Published:** 2025-03-06

**Authors:** DanYang Gu, YuHao Wu, ZhengHua Ding, Yang Dai

**Affiliations:** a Department of General Surgery, Postgraduate Union Training Base of Xiangyang No 1 People's Hospital, School of Medicine, Wuhan University of Science and Technology, Xiangyang City, Hubei Province, China; b Department of General Surgery, Xiangyang No 1 People's Hospital, Hubei University of Medicine, Xiangyang City, Hubei Province, China

**Keywords:** Acute obstructive septic cholangitis, Septic Shock, Bile, HMGB1

## Abstract

**Background:**

Acute Obstructive Septic Cholangitis (AOSC) is a serious infectious disease of the biliary system. It is prone to develop into septic shock without prompt management.

**Methods:**

Bile was collected from 71 AOSC patients (45 AOSC without septic shock, 26 AOSC with septic shock) during biliary drainage and on days 1 and 3 postoperatively. The levels of High Mobility Group Protein 1 (HMGB1), Interleukin (IL)-1, IL-6, and Tumor Necrosis Factor alpha (TNF-α) were measured. The differences in the levels of biliary factors and their correlation with clinical biochemical indicators were assessed in the two groups.

**Results:**

HMGB1 gradually decreased in both groups in the postoperative period. Intraoperative levels of biliary HMGB1 were significantly higher in patients with AOSC with septic shock. TNF-α and HMGB1 decreased slowly in patients with AOSC with septic shock on postoperative days 1 and 3, and the levels of the factors decreased less. Biliary HMGB1 levels were negatively correlated with white blood cell count and positively correlated with blood urea nitrogen, blood creatinine, procalcitonin, and C-reactive protein. A bile HMGB1 level of 1108.3 pg/mL was the cut-off value to differentiate patients with AOSC with or without septic shock.

**Conclusion:**

Biliary HMGB1 levels are elevated in patients with AOSC with septic shock and decrease slowly in the postoperative period. This suggests that HMGB1 is of considerable importance as a potential therapeutic target in the pathogenesis of septic shock in AOSC patients.

## Highlights

• High levels of HMGB1 in AOSC bile are associated with the risk of septic shock.
• AOSC bile HMGB1 is significantly correlated with blood CRP.
• Elevated HMGB1 in AOSC bile increases the risk of postoperative complications.

## Introduction

Acute Obstructive Septic Cholangitis (AOSC), an infectious condition, results from biliary blockage and infections in the biliary tract, representing a critical phase of Acute Cholangitis (AC).^1^ This condition is marked by a sudden suppurative infection and pus accumulation in the biliary system due to blockage, resulting in harm to the hepatobiliary system.^2^ Additionally, the release of substantial quantities of bacteria and toxins into the bloodstream leads to biliary hypertension, widespread organ damage, and ultimately, infectious shock.^3^ The mortality rate of AOSC combined with infectious shock is as high as 20 %‒40 %.^2^ Current treatment of patients with AOSC and septic shock includes antishock and antibiotics, and early relief of obstruction to reduce biliary pressure.^4^ The recently released 2018 Tokyo Guidelines (TG18), updated from TG13, for the diagnosis and grading of the severity of acute cholangitis, are widely accepted and recognized internationally.^5^ In China, the Guidelines for diagnosis and treatment of acute biliary tract infections (2021) were also issued, in which the diagnosis and grading of AC is basically the same as that of TG18.^6^

In the nucleus, the HMGB1 protein binds to chromatin to form chromatin-binding complexes.^7^ Recent studies have determined that HMGB1, when released into the extracellular environment, acts as a mediator of inflammation to stimulate and activate the immune system.^8^^,^^9^ As a response to infection, HMGB1 acts as a pro-inflammatory cytokine that may lead to cell and tissue damage.^10^ As a result of this process, metabolic acidosis, multiple organ dysfunction syndrome, and sepsis can develop.^11^ Serum HMGB1 levels have been shown to recognize infectious diseases.^12^ Moreover, it has been shown to be a potent pro-inflammatory factor in sepsis.^13^ Recent studies have reported the possibility of HMGB1 as a therapeutic target for sepsis.^14^^,^^15^ In addition, a previous study demonstrated the association of HMGB1 with sepsis in serum and cells of AOSC patients, with HMGB1 levels progressively decreasing with treatment.^16^ Currently, it is widely accepted that severe or fatal sepsis is mediated by inflammatory mediators such as Interleukin (IL)−1, IL-6, and Tumor Necrosis Factor (TNF)-α. All of these factors have an important role in the initiation, maintenance, and resolution of inflammation.^17^^,^^18^ AOSC not only causes cholestatic bile duct obstruction but also leads to increased bacteria and inflammatory factor production. Plasma and biliary inflammatory factor levels are increased in patients with AC and correlate with disease severity.^19^ The present study hypothesized that septic shock in patients with AOSC is associated with increased levels of proinflammatory factors and that these factors are related to the clinical biochemical characteristics of the patients.

## Materials and methods

### Patients

This study followed STARD guidelines. Seventy-one patients with AOSC diagnosed and treated at Xiangyang No 1 People's Hospital, Hubei University of Medicine from January 2022 to December 2023 were prospectively analyzed. AOSC diagnosis met the diagnostic criteria of the TG18 for severe cholangitis (Grade III).^5^ The diagnosis of septic shock was judged according to the criteria of the Guidelines for diagnosis and treatment of acute biliary tract infections (2021) and the Chinese Guidelines for Emergency Treatment of Sepsis/Septic Shock (2018).^6^^,^^20^ Of the 71 patients with AOSC, 26 patients had comorbid septic shock (i.e., mean arterial pressure 〈 65 mmHg and blood lactate concentration 〉 2 mmoL/L despite adequate fluid resuscitation). The patients with comorbid septic shock ended up with 8 fatal cases. Written informed consents were not provided because it is a retrospective study. All the records were processed anonymously and personal identifications were canceled before analyzing.

Inclusion criteria: 1) Age above 18 years; 2) Compliance with the diagnosis of AOSC and sepsis. Exclusion criteria: 1) Pregnant and lactating women; 2) Cardiovascular system dysfunction, consciousness disorder, respiratory disorder, hepatic dysfunction, hematologic dysfunction, and renal dysfunction caused by non-AOSC; 3) Bile duct stenosis or obstruction due to malignancy; 4) Infectious shock caused by non-AOSC; 5) Incomplete clinical data.

Of the 71 patients, 43 had common bile duct stones, 5 intrahepatic bile duct stones, 13 combined intrahepatic and extrahepatic bile duct stones as well as 10 bile duct stones combined with benign biliary stricture. The patients underwent biliary drainage including endoscopic retrograde cholangiopancreatography, percutaneous transhepatic catheter drainage, endoscopic sphincterotomy, or open surgery within 24 h of diagnosis. Information on postoperative complications of patients was obtained, including biliary pancreatitis, incision infections, pulmonary infections, sepsis/septic shock and death.

### Baseline data collection and AOSC diagnosis

Data on patients' gender, age, Body Mass Index (BMI), blood pressure, and etiology were collected through the medical record system.

Severe cholangitis (Grade III) at least met any one of the following 1) Cardiovascular system dysfunction: Requirement for 5 g/kg/min^-1^ of dopamine, or maintenance of hypotension with any dose of norepinephrine. 2) Neurologic dysfunction: an impairment of consciousness. 3) Respiratory dysfunction: PaO_2_/FiO_2_ < 300 mmHg. 4) Abnormal hepatic function: PT-INR > 1.5. 5) Renal dysfunction: Oliguria, SCr > 2 mg/dL. 6) Hematologic dysfunction: Platelets < 100 × 10^9^/L.

### Blood collection and related indicator tests

Participants fasted for at least 3 h before blood collection. Blood samples were collected in vacuum tubes with or without EDTA K2 anticoagulant. The anticoagulant-containing samples were then centrifuged at 4 °C, 3000 rpm for 15 min, and then the supernatant was centrifuged for another 10 min. The sample without anticoagulant was left at 4 °C for 2 h and then centrifuged at 3000 rpm for 20 min. White Blood Cells (WBC), Platelet count (PKT), Hemoglobin (Hb), albumin, total bilirubin, direct bilirubin, Aspartate Aminotransferase (AST), Alanine Transaminase (ALT), γ-Glutamyl Transpeptidase; (γ-GT), Alkaline Phosphatase (ALP), C-Reactive Protein (CRP), Blood Urea Nitrogen (BUN), Serum Creatinine (SCr), and Prothrombin Time (PT) using standard laboratory methods.

### Bile collection and cytokine measurement

Bile (5 mL) was extracted intraoperatively and 1 and 3 days postoperatively, respectively. The sample was centrifuged for 15 min at 4 °C and 3000 r/min, and the supernatant was taken and stored at −80 °C. Interleukin (IL)−1, IL-6, Tumor Necrosis Factor-α (TNF-α), and HMGB1 (R and D Systems, MN, USA) in bile supernatant and serum were determined by Enzyme-Linked Immunosorbent Assay (ELISA). Subsequently, assays were performed using the Ella fully automated ultrasensitive ELISA system (ProteinSimple, USA).

### Data analysis

Estimation of the study's sample size was conducted using G*Power software version 3.1.9.2, applying a significance threshold of α = 0.05, a power range of 1-β = 0.8, an effect size of d = 0.5, followed by bilateral testing. The statistical evaluations were conducted utilizing the SPSS 20.0 software. The frequency-based count data underwent analysis through the Chi-Square test or Fisher's exact test. The Shapiro-Wilk test was employed to verify the normality of the data. Every piece of continuous data was presented as mean ± standard deviation and was analyzed using Student's *t*-tests for normal distribution and Mann-Whitney U *tests* for skewed distribution. The study employed bivariate correlation analyses for individuals, complemented by Bonferroni correction. The Receiver Operating Characteristic Curves (ROC) were graphically represented, and the area beneath these curves (AUC) was determined. The charts were created utilizing GraphPad Prism 8.

## Results

### Patients’ general characteristics and experimental measurements

Table 1 shows the general characteristics and experimental measurements of the patients in both groups. There were no significant differences in gender, age, and BMI between the two groups. On blood routine, WBC, and PLT differed between the two groups, and patients in the AOSC with septic shock group had higher levels (both *p* < 0.05). Differences in liver function were not significant in patients with septic shock and patients without septic shock (both *p* > 0.05). BUN and SCr levels were higher in patients with septic shock than in patients without septic shock (both *p* < 0.05). CRP and PCT were significantly different between the two groups, with higher levels in AOSC patients with septic shock (both *p* < 0.05). No difference in prothrombin time was observed between the two groups (*p* > 0.05).

**Table 1:** General characteristics of patients and intraoperative clinical indicators.

	**AOSC without septic shock**	**AOSC with septic shock**	**p-value**
	**(*n* = 45)**	**(*n* = 26)**	
Age (years)	60.36 ± 10.36	57.25 ± 11.07	0.521
Gender (Female/Male)	20/25	12月14日	0.889
BMI (kg/m2)	23.85 ± 2.12	24.25 ± 1.79	0.432
WBC (×109/L)	18.25 ± 5.38	20.85 ± 4.35	0.041
PLT (g/L)	135 ± 46.25	89 ± 35.36	< 0.001
Hb (g/dL)	11.82 ± 0.45	11.45 ± 0.32	0.746
Albumin (g/dL)	3.02 ± 0.19	2.95 ± 0.21	0.452
Total bilirubin (mg/dL)	3.62 ± 0.38	3.71 ± 0.42	0.618
Direct bilirubin (mg/dL)	2.56 ± 0.46	2.65 ± 0.49	0.746
AST (U/L)	198.36 ± 42.36	200.35 ± 36.31	0.846
ALT (U/L)	157.46 ± 27.46	159 ± 26.72	0.942
γ-GT (U/L)	386.46 ± 50.26	372.48 ± 48.54	0.246
ALP (U/L)	445.16 ± 153.62	465.15 ± 164.15	0.327
Amylase (U/L)	79.65 ± 10.25	76.49 ± 11.59	0.482
BUN (mg/dL)	24.24 ± 1.26	26.78 ± 1.37	<0.001
SCr (mg/dL)	1.41 ± 0.24	1.53 ± 0.18	<0.001
PCT (ng/mL)	4.30 ± 1.21	4.91 ± 1.07	0.046
CRP (mg/dL)	9.89 ± 1.25	11.28 ± 1.14	<0.001
Prothrombin time (sec)	79.1 ± 2.6	80.4 ± 2.4	0.846

BMI, Body Mass Index; WBC, White Blood Cells; PLT, Platelet Count; Hb, Blood Hemoglobin; AST, Aspartate Aminotransferase; ALT, Alanine Transaminase; γ-GT, γ-Glutamyl Transferase; BUN, Blood Urea Nitrogen; SCr, Serum Creatinine; ALP, Alkaline Phosphatase; CRP, C‑Reactive Protein. Count data were expressed as frequency and compared using the pearson Chi-Square test; all continuous data were expressed as mean ± standard deviation and compared with Student's *t*-test.

### Bile factor levels in patients

**Fig. 1:**
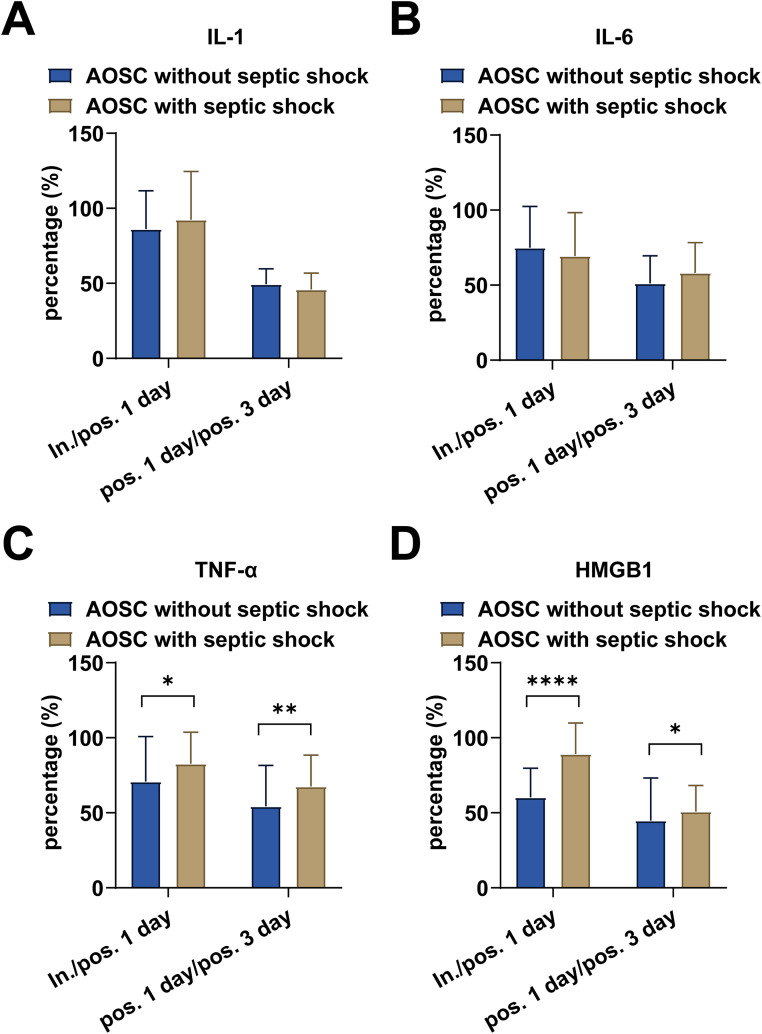
**Comparison of postoperative reduction in biliary factor levels in two groups of patients.** (A) IL-1; (B) IL-6; (C) TNF-α; (D) HMGB1. Results are expressed as mean ± SD, and Student's *t*-test or Mann-Whitney U *test* was used for comparison. * *p* < 0.05, ** *p* < 0.01, *** *p* < 0.001. p-value < 0.05 is statistically different.

Table 2 shows the intraoperative and postoperative bile factor levels on the 1st and 3rd postoperative days. HMGB1 was significantly different intraoperatively between the two groups, with patients with septic shock having higher levels (*p* < 0.05), whereas there were no statistically significant differences in the levels of IL-1, IL-6, and TNF-α (all *p* > 0.05). IL-1, IL-6, TNF-α, and HMGB1 were in a continuous decrease postoperatively in both groups relative to intraoperative. This suggests that these factors can be progressively reduced after receiving treatment, which may indicate the subsidence of inflammation in patients. Of interest, TNF-α and HMGB1 in the bile of patients with septic shock were higher than those of patients without septic shock postoperatively. To further observe the extent to which these factors were reduced in the postoperative period, the percentage reduction in biliary factors was compared in the postoperative period, as shown in Fig. 1. Interestingly, the percentage reduction in TNF-α and HMGB1 levels was lower in patients with septic shock than those without septic shock (both *p* < 0.05), while IL-1 and IL-6 were similarly lowered between the two groups, with no statistically significant difference (both *p* > 0.05). This suggests that high levels of biliary HMGB1 intraoperatively are associated with the risk of sepsis in AOSC patients and that it's having a delayed decrease in postoperative bile may be associated with systemic inflammatory response syndrome due to septic infection.

**Table 2:** Comparison of intraoperative and postoperative factor levels in bile of patients.

	**AOSC without septic shock (*n* = 45)**			**AOSC with septic shock (*n* = 26)**		
	**In.**	**Pos. 1 day**	**Pos. 3 day**	**In.**	**Pos. 1 day**	**Pos. 3 day**
**IL-1 (pg/mL)**	887.65 ± 189.32	727.36 ± 102.41^b^	352.41 ± 56.35^c^	900.52 ± 124.65	775.46 ± 112.5^b^	348.55 ± 52.78^c^
**IL-6 (pg/mL)**	754.65 ± 214.72	521.45 ± 105.34^b^	257.47 ± 57.15	770.92 ± 206.45	486.42 ± 99.49^b^	267.15 ± 54.36^c^
**TNF-α (pg/mL)**	614.34 ± 145.83	412.25 ± 124.14^b^	206.36 ± 74.52	598.47 ± 91.71	483.38 ± 105.43^a^^,^^b^	316.59 ± 66.18^a^^,^^c^
**HMGB1 (pg/mL)**	811.42 ± 180.49	469.24 ± 115.72^b^	193.15 ± 91.18	1026.45 ± 238.14^a^	882.25 ± 105.42^a^^,^^b^	426.64 ± 68.48^a^^,^^c^

Results are expressed as mean ± standard deviation, and the Student's *t*-test was used for between-group comparisons.

a *p* < 0.05, AOSC with septic shock group vs. AOSC without septic shock group.

b *p* < 0.05 vs. intraoperative in the same group.

c *p* < 0.05 vs. Pos. 1 day in the same group; p-value < 0.05 statistically different.

### Correlation of intraoperative bile factors with clinical biochemical indices

Subsequently, the authors used bivariate analysis to correlate these intraoperative bile factors in AOSC patients with clinical biochemical indices that were statistically different between the two groups, including WBC, PLT, BUN, SCr, PCT, and CRP, as shown in Table 3. PTL correlated with each factor with a negative correlation (all *p* < 0.05). IL-1 had weak positive correlations with PCT and CRP (all *p* < 0.05); IL-6 had a weak positive correlation with BUN (*p* < 0.05); TNF-α had weak positive correlations with SCr, PCT, and CRP (all *p* < 0.05); and except for WBC, HMGB1 had positive correlations with all other biochemical indices, and had a moderate correlation with CRP (all *p* < 0.05). These results suggest that intraoperative bile factors, especially HMGB1, are associated with clinical biochemical indices in AOSC patients.

**Table 3:** Correlation analysis between intraoperative bile factors and clinical biochemical parameters.

	**IL-1**	**IL-6**	**TNF-α**	**HMGB1**
**WBC**	0.158	0.048	0.149	−0.094
**PLT**	−0.315^a^	−0.179^a^	−0.345^a^	−0.495^a^
**BUN**	0.147	0.175^a^	0.156	0.245^a^
**SCr**	0.104	0.167	0.256^a^	0.351^a^
**PCT**	0.185^a^	0.152	0.297^a^	0.302^a^
**CRP**	0.235^a^	0.106	0.341^a^	0.512^a^

Person correlation analysis was used, and correlation coefficients *r* are indicated. All p-values were Bonferroni corrected. Corrected *p* < 0.05 is statistically significant.

a p-adjust. < 0.05. *r* = 0.1‒0.4, weak correlation; *r* = 0.4‒0.6, moderately strong correlation.

### Efficacy of HMGB1 to assess AOSC with septic shock

**Fig. 2:**
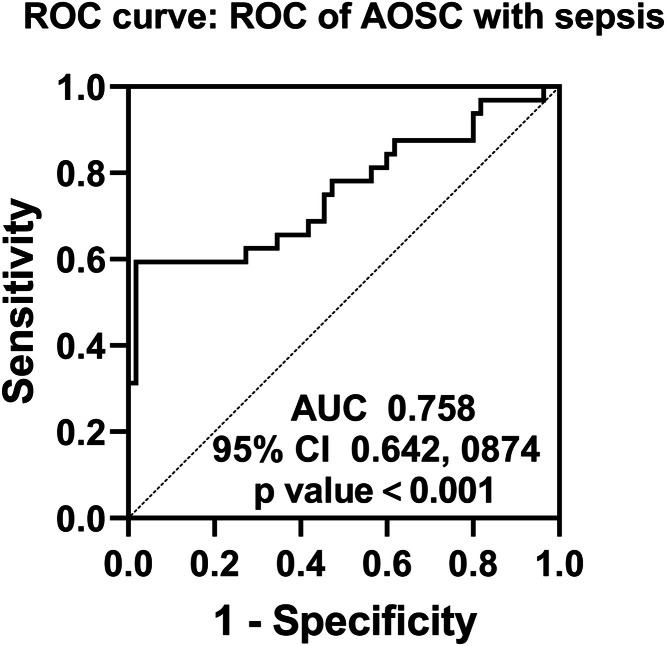
**ROC curve.** AUC was calculated to assess the value of intraoperative biliary HMGB1 to differentiate AOSC with or without septic shock.

Initially, patients were stratified into subgroups according to the third quartile of their intraoperative bile HMGB1 levels to examine the association between HMGB1 concentrations and postoperative complications and mortality, as detailed in Table 4. The subgroup with elevated bile HMGB1 levels exhibited a higher incidence of complications, including 7 cases of biliary pancreatitis, 19 cases of sepsis or septic shock, and 7 cases of mortality. Among the 71 patients, 9 succumbed, with septic shock accounting for 8 of these fatalities. Subsequently, based on this, the authors plotted ROC curves and AUC to assess the value of bile HMGB1 in differentiating AOSC with or without septic shock, as shown in Fig. 2. Bile HMGB1 was effective in differentiating AOSC with or without septic shock with an AUC of 0.758 (95 % CI 0.642‒0.874). The optimal cutoff value was 1108.3 pg/mL with a sensitivity and specificity of 60.34 % and 96.36 %, respectively. These results suggest that intraoperative bile HMGB1 can better differentiate patients with AOSC with or without septic shock, favoring HMGB1 as a target for potential treatment of septic shock occurring in patients with AOSC.

**Table 4:** The impact of HMGB1 levels on postoperative complications and mortality.

	**<246.03 pg/mL (*n* = 23)**	**246.03 ∼ 1050.31 (*n* = 25)**	**>1050.31 pg/mL (*n* = 23)**	***p*-value**
**Biliary pancreatitis**	0 (0.00)	2 (8.00)	7 (30.43)	0.005
**Incision infection**	1 (4.35)	2 (8.00)	1 (4.35)	1.000
**pulmonary infection**	0 (0.00)	1 (4.00)	3 (13.04)	0.206
**Sepsis/septic shock**	1 (4.35)	6 (24.00)	19 (82.61)	<0.001
**Death**	0 (0.00)	2 (8.00)	7 (30.43)	0.005

The data was displayed as n (%), and Fisher's exact test is used for comparison; *p* < 0.05 has statistical significance.

## Discussion

AC manifests as a widespread infectious disorder, marked by sudden inflammation and biliary tract infections. AC stems from bacteria settling in the bile ducts, elevating their internal pressure and causing a reflux of bacteria and endotoxins in the bloodstream. AOSC represents an intense type of cholangitis characterized by the accumulation of pus in the bile ducts.^21^ Although TG18 complements TG13 with an AC treatment approach, it has a greater emphasis on surgery and incorporates an assessment of surgical risk. AOSC that is not promptly resolved can progress further to septic shock.^22^ Markers for the diagnosis of AOSC-induced septic shock would provide a useful step in the treatment of AOSC combined with septic shock. Recently, several studies have identified HMGB1 as a target for the diagnosis and treatment of sepsis.^13^^,^^23^ The present study focused on the correlation of HMGB1 of bile with disease severity and with clinical biochemical parameters in AOSC patients.

Septic shock has become one of the major causes of death in clinically critical patients. The root cause is the excessive release of cytokines and inflammatory mediators, forming a pro-inflammatory cytokine response network, leading to uncontrolled inflammatory response and immune dysfunction.^24^ HMGB1 is “ate inflammatory mediator” in sepsis, and it is the center of the pro-inflammatory cytokine response network in sepsis.^25^^,^^26^

The present study showed that intraoperative levels of HMGB1 were higher in patients with AOSC with septic shock than in patients with AOSC. After drainage, HMGB1, IL-1, IL-6, and TNF-α in the bile of AOSC patients were reduced as infection decreased. Recent studies have confirmed that extracellular HMGB1 acts as a late mediator mediating delayed toxin lethal shock.^27^ Similar results were found in this study, where biliary HMGB1 levels were not only higher intraoperatively in patients with AOSC with septic shock than in patients without septic shock but also declined more gently and to a lesser extent than in patients with AOSC with septic shock on postoperative days 1 and 3. There are two possible mechanisms regarding the role of HMGB1 as a delayed inflammatory factor mediating inflammation: 1) Direct toxic effects, with experiments showing that direct injection of recombinant A box protein (which has the function of targeting the inhibition of HMGB1) markedly reduces recombinant HMGB1-mediated lethality.^28^ 2) Interaction with proinflammatory factors (Inflammatory cytokines stimulate the release of HMGB1). Literature reports have found that HMGB1 is formed by macrophages and monocytes in intracellular HMGB1-rich vesicles after stimulation by IL-1 and TNF-α, and the cells then release HMGB1 extracellularly by cytosolization.^29^ At the same time, HMGB1 also stimulates the release of inflammation-causing factors from macrophages and monocytes, such as IL-1, IL-6, IL-8, and TNF-α, which in turn further lead to worsening inflammation severity.^30^ HMGB1 in monocyte cultures significantly stimulates IL-1, IL-6, and TNF-α, among others.^31^ The authors only observed higher levels of HMGB1 in patients with AOSC with septic shock, and we did not find any differences in the intraoperative levels of other inflammatory factors IL-1, IL-6, and TNF-α between the two groups. However, TNF-α and HMGB1 levels were higher in AOSC with septic shock patients than in AOSC patients at 1 and 3 days postoperatively, and the slow postoperative reduction of TNF-α and HMGB1 was also confirmed by calculating the degree of decline. These results suggest that septic shock in AOSC patients may be due to a greater role of HMGB1-mediated direct toxicity; in the postoperative period, HMGB1 maintains and prolongs the inflammatory response mainly in combination with TNF-α. However, more relevant studies will have to be conducted to confirm this.

PLT is associated with bloodstream infections in patients with sepsis and septic shock, and a reduced PLT has predictive value.^32^ The present study also showed that PLN was reduced in patients with AOSC combined with septic shock. Septic shock leads to multi-organ damage. Biliary obstruction is associated with an increased risk of hepatic failure.^33^ However, in this study, AST, ALT, γ-GT, and ALP, which reflect the damage to liver tissues, and total bilirubin and direct bilirubin, which reflect the function of hepatic bile production and metabolism, did not differ significantly between the two groups of patients at the time of admission to the hospital. Therefore, it is necessary to further evaluate the liver function of patients after septic shock. Of note, there was a difference in renal function between the two groups at the time of admission, with patients with septic shock having significantly higher BUN and SCr levels than AOSC patients without septic shock. Sepsis or septic shock is considered a major contributing factor to injury.^34^ Therefore, the authors believe that renal injury in AOSC patients contributes more significantly to septic shock. PCT is a protein whose plasma levels are elevated in severe bacterial, fungal, and parasitic infections, as well as in sepsis and multiple organ failure.^35^ Hepatocytes produce CRP in response to inflammatory stimuli such as microbial infection or injury to acute phase proteins in the body.^36^ PTC and CRP have been shown to be diagnostic of septic shock and correlate with the risk of patient death.^37^ Additionally, postoperative adverse outcomes based on subgroups of intraoperative HMGB1 levels suggested that biliary pancreatitis, sepsis, and death were associated with higher HMGB1 levels in bile during surgery. The authors observed that biliary HMGB1 levels were negatively correlated with PLT, positively correlated with BUN and SCr, and positively correlated with PCT and CRP. Finally, bile HMGB1 1108.3 pg/mL was the cutoff value to differentiate patients with AOSC with or without septic shock.

### Limitations

The main limitations of this study are the single-center design and the advanced age of the patients. Therefore, the results of this study only support the characterization of biliary HMGB1 levels in study participants in the same or similar settings. In addition, the sample size was small, which may limit the generalizability of the findings to the global population and other age groups. There is a need for multicenter studies with expanded sample sizes to further investigate the independent risk factors for septic shock in patients with AOSC.

## Conclusion

In this study, the authors confirmed elevated levels of HMGB1 in the bile of patients with AOSC with septic shock. In addition, the authors found a correlation between biliary HMGB1 levels and clinical biochemical indicators that characterize the severity of the disease, as well as an interaction between it and TNF-α. In addition, biliary HMGB1 declined slowly postoperatively in patients with AOSC with septic shock, suggesting a role for biliary HMGB1 in maintaining the inflammatory response postoperatively. Together, these findings suggest the potential value of biliary HMGB1 levels as a biomarker of septic shock in AOSC.

## Data available

Data is available from the corresponding author on request.

## Ethics statement

Not applicable. This retrospective study did not require ethical board approval.

## Informed consent

Written informed consents were not provided because it is a retrospective study. All the records were processed anonymously, and personal identifications were canceled before analyzing.

## Authors’ contributions

Conceptualization, DanYang Gu and YuHao Wu; Methodology, ZhengHua Ding and Yang Dai; Formal analysis, YuHao Wu and ZhengHua Ding; Investigation, DanYang Gu and Yang Dai; Data curation, DanYang Gu and YuHao Wu; Writing-original draft preparation, DanYang Gu and YuHao Wu; Writing-review and editing, Yang Dai. All authors have read and agreed to the published version of the manuscript.

## Funding

This project is supported by the Faculty Development Grants of Xiangyang No.1 People's Hospital Affiliated to Hubei University of Medicine (XYY2023D01) and the Xiangyang City Medical and Health Science and Technology Project (nº 2022YL25A).

## Declaration of competing interest

The authors declare no conflicts of interest.
